# Case report: A case of epidermolysis bullosa acquisita with IgG and IgM anti-basement membrane zone antibodies relapsed after COVID-19 mRNA vaccination

**DOI:** 10.3389/fmed.2023.1093827

**Published:** 2023-05-30

**Authors:** Satoko Minakawa, Yasushi Matsuzaki, Shogo Yao, Chihiro Sagara, Eijiro Akasaka, Hiroshi Koga, Norito Ishii, Takashi Hashimoto, Daisuke Sawamura

**Affiliations:** ^1^Department of Dermatology, Hirosaki University Graduate School of Medicine, Aomori, Japan; ^2^Department of Clinical Laboratory, Hirosaki University Hospital, Aomori, Japan; ^3^Department of Dermatology, Kurume University School of Medicine, Fukuoka, Japan; ^4^Department of Dermatology, Osaka Metropolitan University Graduate School of Medicine, Osaka, Japan

**Keywords:** autoimmune blistering diseases, EBA, immunofluorescence, enzyme-linked immunosorbent assay (ELISA), type VII collagen

## Abstract

We report a case of autoimmune bullous disease (AIBD) with IgG and IgM autoantibodies against epidermal basement membrane zone (BMZ), which showed recurrence of mucocutaneous lesions after coronavirus disease 2019 (COVID-19) mRNA vaccination. A 20-year-old Japanese woman with a 4-year history of epidermolysis bullosa acquisita (EBA) presented to our clinic. She noticed fever and rash on the same day and visited at our hospital 2 days later. Physical examination revealed blisters, erosions and erythema on the face, shoulder, back, upper arms, and lower lip. A skin biopsy from the forehead showed subepidermal blister. Direct immunofluorescence showed linear depositions of IgG, IgM, and C3c in the epidermal BMZ. By indirect immunofluorescence of 1M NaCl-split normal human skin, circulating IgG autoantibodies were bound to the dermal side of the split at 1:40 serum dilution, and circulating IgM antibodies were bound to the epidermal side of the spilt. After the increase of prednisolone dose to 15 mg/day, the mucocutaneous lesions resolved in a week. The present case is the first case of possible EBA with IgG and IgM anti-BMZ antibodies, in which the mucocutaneous lesions were recurred after COVID-19 mRNA vaccination. Clinicians should be aware that bullous pemphigoid-like AIBDs, including EBA and IgM pemphigoid, might be developed after COVID-19 mRNA vaccination.

## Introduction

Autoimmune bullous diseases (AIBDs), a group of tissue specific autoimmune disease of the skin, are classified into various subtypes with the different immunoglobulin types and autoantigens (1). Epidermolysis bullosa acquisita (EBA) is characterized clinically by blisters and scars of the skin and erosive mucosal lesions, and immunologically by IgG autoantibodies against type VII collaegen (2, 3). In many EBA cases, oral administration of corticosteroids are effective but do not lead to complete remission.

AIBDs have been reported to be induced or exacerbated after immunization with various vaccines, including vaccines for measles, varicella zoster, influenza, hepatitis B and human papillomavirus (4).

The rapid flare of a bullous diseases after vaccination is not novel. Bullous pemphigoid (BP) has been reported to occur within 24 hours after vaccination (5). In addition, among the 12 patients who newly developed subepidermal blistering lesions after the first or second coronavirus disease 2019 (COVID-19) mRNA vaccination, the diagnosis of BP was confirmed in eight patients by the results of direct immunofluorescence (DIF), indirect immunofluorescence (IIF) of 1 M NaCl-split skin and/or enzyme-linked immunosorbent assays (ELISA) of BP180 (6).

In this report, we present with a case of EBA with IgG and IgM autoantibodies against epidermal basement membrane zone (BMZ), which showed recurrence of mucocutaneous lesions 2 days after COVID-19 mRNA vaccination.

## Case report

**Figure 1:**
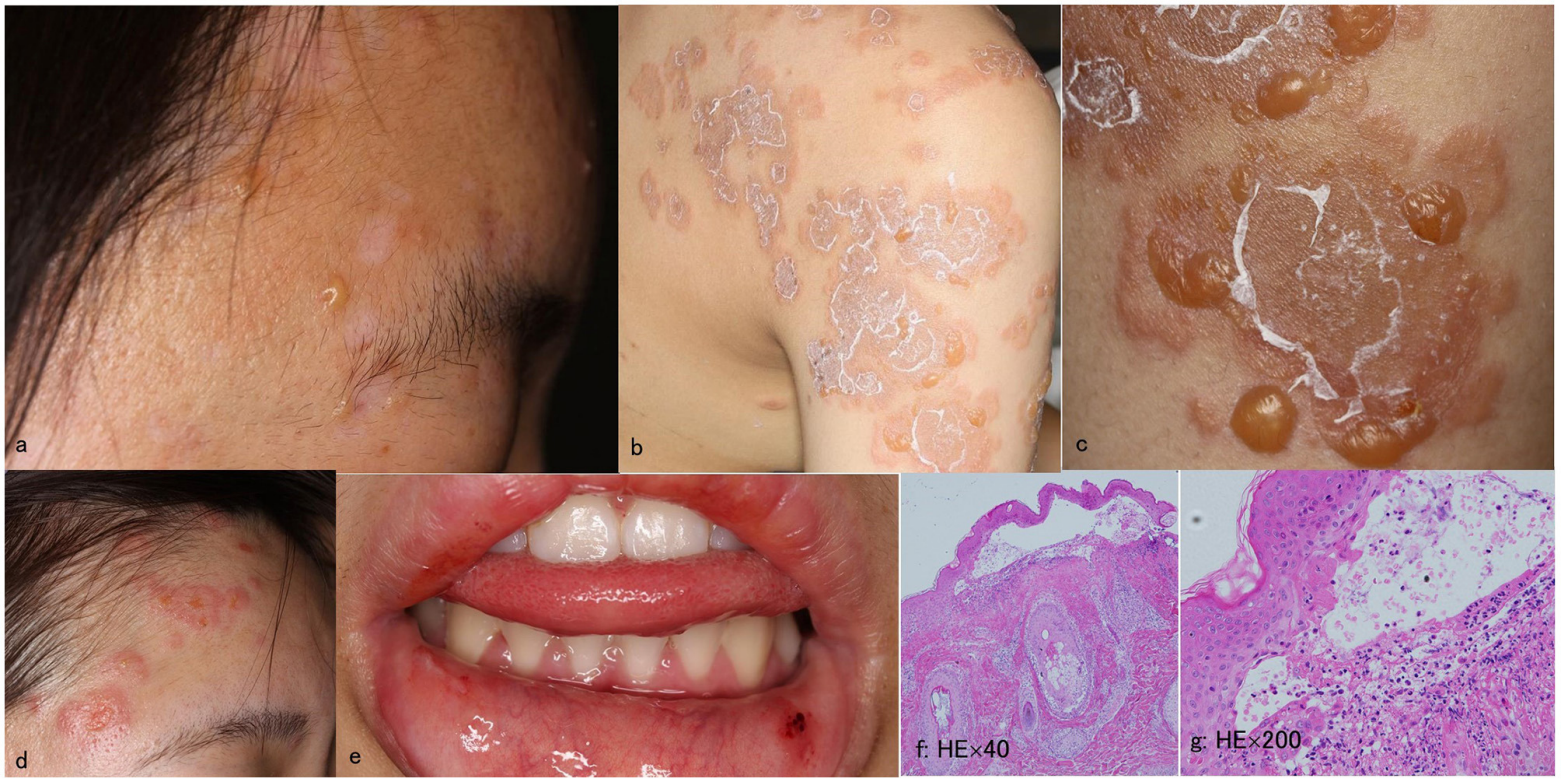
Clinical and histopathological features at the first visit and at the time of relapse. **(a–e)** Clinical features of blisters, erosions, and erythema on the forehead **(a)**, shoulder **(b)**, and upper arms **(c)** at the first visit, and those on the face **(d)**, and lower lip **(e)** at the time of relapse. **(f, g)** Histopathology of the skin biopsy from the forehead at the time of relapse showing subepidermal blisters with infiltration of neutrophils in the blister and dermis [original magnifications; **(f)** ×40 and **(g)** ×200].

A 16-year-old Japanese female presented with a 1-month history of erosions on the lips. She had no medical history. Physical examination revealed blisters, erosions and erythema on the face (Figure 1a), lips, back, shoulders (Figure 1b) and arms (Figure 1c). Histopathology for a skin biopsy from the face showed subepidermal blisters. DIF showed linear depositions of IgG, IgM, and C3c in the epidermal BMZ (data not shown).

**Figure 2:**
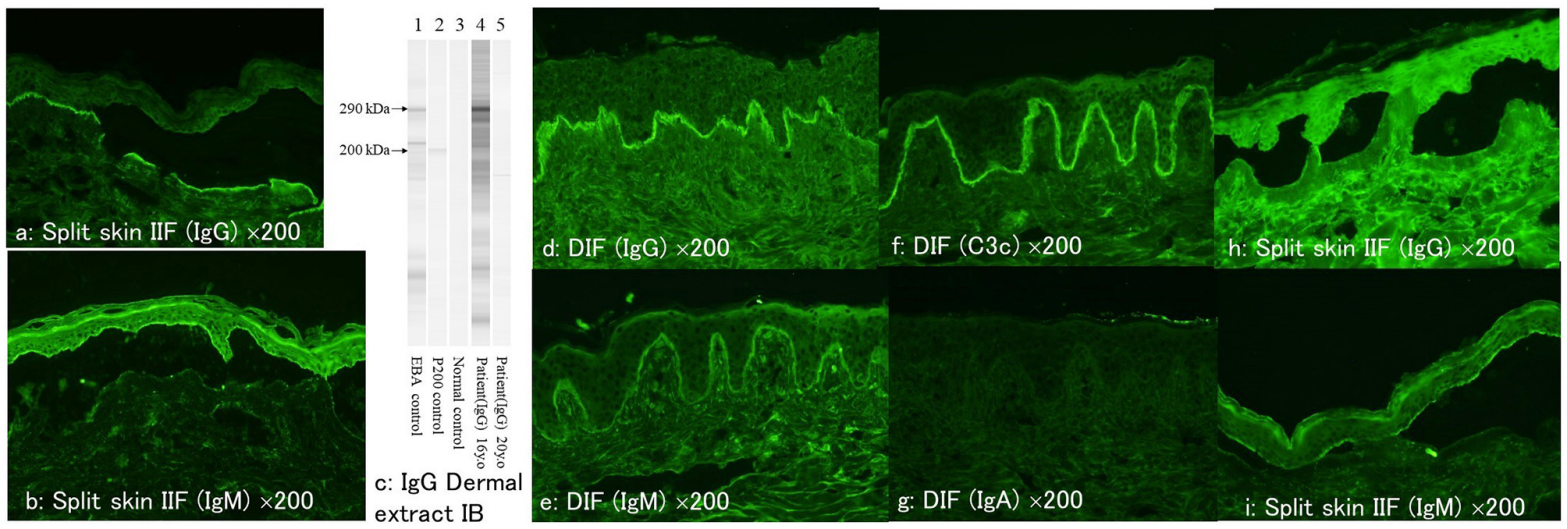
The result of various immunological tests at the first visit and at the time of relapse. **(a, b)** At the first visit, indirect immunofluorescence of 1M NaCl-split skin showing positive reactivity with dermal side for IgG antibodies **(a)** and with epidermal side for IgM antibodies **(b)**. **(c)** The results of IgG immunoblotting of normal human dermal extract. The serum at the first visit (lane 4), but not the serum at the relapse (lane 5), reacted with the 290 kDa type VII collagen. **(d–g)** Direct immunofluorescence at the time of relapse showing positive results for IgG **(d)**, IgM **(e)**, and C3c **(f)** but negative for IgA **(g)**. **(h, i)** At the time of relapse, indirect immunofluorescence of 1M NaCl-split skin showing positive reactivity with dermal side for IgG antibodies **(h)** and with epidermal side for IgM antibodies **(i)** (all of the immunofluorescence figures: original magnification, ×200).

Circulating IgG anti-BMZ autoantibodies were detected by IIF of normal human skin, which bound to the dermal side of 1 M NaCl-split normal human skin at 1:40 serum dilution (Figure 2a). Circulating IgM autoantibodies were bound to the epidermal side of 1 M NaCl-split normal human skin at 1:40 serum dilution (Figure 2b). IgG ELISAs (MBL, Japan) showed positive results for type VII collagen (index 39.76; cut-off < 6.14), but negative for desmoglein 1, desmoglein 3, BP180 and BP230. Immunoblotting of normal human dermal extract detected IgG antibodies to type VII collagen (Figure 2c), while IgG immunoblotting analyses of other substrates were negative. IgM immunoblotting analyses of normal human epidermal extracts, recombinant proteins of NC16a and C-terminal domains of BP180 and concentrated culture supernatant of HaCaT cells were negative.

The diagnosis of EBA with IgM antibodies to unknown antigen was made, and the initiation of oral prednisolone 30 mg/day gradually improved the skin and oral mucosal symptoms. However, the skin lesions were intaractable, and the patient stayed on prednisolone for the 4 years until the recurrence, when the patient was treated with prednisolone 2 mg/day.

Four years later, after the patient received first dose of COVID-19 mRNA vaccination, she first noticed fever on day 1 on the same day and then bullous skin lesions 2 days later. Then the patient visited at our hospital. Physical examination revealed blisters, erosions and erythema on the face (Figure 1d), shoulder, back, upper arms, and lower lip (Figure 1e). A skin biopsy from the forehead histopathologically showed subepidermal blisters (Figures 1f, g). DIF showed linear depositions of IgG, IgM, and C3c, but not IgA, in the epidermal BMZ (Figures 2d–g).

By IIF of 1M NaCl-split normal human skin, circulating IgG autoantibodies were bound to the dermal side of the split at 1:40 serum dilution (Figure 2h), and circulating IgM antibodies were bound to the epidermal side of the spilt (Figure 2i). IgG type VII collagen ELISA was negative (index 5.78; cut-off < 6.14). Immunoblotting of normal human dermal extract did not detect IgG antibodies to type VII collagen (Figure 2c). IgM immunoblotting analyses using the 4 substrates were negative. The results of various immunological tests both at the first visit 4 years before and after the COVID-19 mRNA vaccination were summarized in Table 1.

**Table 1:** The summary of the results of various immunological tests both at the first visit 4 years before and at the time of relapse after the COVID-19 vaccination.

	**First visit (16 year old )**	**Relapse (20 year old)**
DIF (BMZ) IgG	(+)	(+)
DIF (BMZ) IgA	(–)	(–)
DIF (BMZ) IgM	(+)	(+)
DIF (BMZ) C3c	(+)	(+)
IIF (BMZ) IgG	(+) × 40	(–)
IIF (BMZ) IgM	(–)	(–)
ss-IIF (epidermis) IgG	(–)	(–)
ss-IIF (epidermis) IgM	(+) × 10	(+) × 40
ss-IIF (dermis) IgG	(+) × 40	(+) × 40
ss-IIF (dermis) IgM	(–)	(–)
IgG Dermal extract IB: 290 kDa	Pos	Neg
IgG Dermal extract IB: 200 kDa	Neg	Neg
IgG Dermal extract IB: laminin 332	Neg	Neg
IgG ELISAs type VII collagen (cut-off Index < 6.14)	39.76	5.78
Anti-BP180 NC16A IgG ELISA	Neg	Neg
Anti-BP230 IgG ELISA	Neg	Neg

BMZ, basement membrane zone; DIF, direct immunofluorescence; IB, immunoblotting; IIF, indirect immunofluorescence; Neg, negative; Pos; positive; ss-IIF, 1M NaCl split skin indirect immunofluorescence; (+), linear deposits of immunoreactants; (–), no deposits of immunoreactants.

After the increase of prednisolone dose form 2 to 15 mg/day, the mucocutaneous lesions resolved in a week.

## Discussion

COVID-19 mRNA vaccination has been reported to induce not only AIBDs but also many other skin conditions (7). From the positive results in DIF and IIF analyses, as well as the clinical features of prominent blisters, we considered that the present case might have had a relapse of EBA by COVID-19 mRNA vaccination 4 years after the first visit, although the 2 days may not be too short for the recurrence of autoimmune disease, and we could not detect specific autoantigens at this recurrent time.

Regarding the precise diagnosis for the present case, at the first visit, the results of various immunological tests, including positive IgG reactivity with type VII collagen, made the diagnosis of EBA, although IgM anti-BMZ antibodies co-existed.

The mucocutaneous lesions recurred after COVID-19 mRNA vaccination were most likely the recurrence of EBA from the positive IgG reactivity with dermal side of 1 M NaCl-split skin, although we could not detect IgG reactivity with type VII collagen. The reason for the failure to detect anti-type VII collagen might be either that the antibody titer was too low or that the epitope on type VII collagen had changed. The possibility of the change from EBA to the other AIBDs with IgG reactivity with dermal side, i.e., anti-laminin 332-type mucous membrane pemphigoid (MMP) or ant-p200 pemphigoid, is less conceivable.

Recently, Hirano et al. (8) proposed IgM pemphigoid, which showed anti-epidermal BMZ antibodies exclusive of IgM type, as a novel AIBDs entity. However, the disease entity of IgM pemphigoid has not been widely accepted. The clinical and histopathological characteristics, as well as the therapies and disease course, in IgM pemphigoid remain unknown. IgM autoantibodies in IgM pemphigoid tend to react with non-NC16A domain of BP180, probably C-terminal domain (8). Another possibility is that this case had IgM pemphigoid, and IgM anti-BMZ antibodies caused the mucocutaneous lesions both at the onset and at the time of relapse.

An older study suggested that IgM deposits (granular and linear) are present in the sun-exposed skin of healthy adults (*n* = 10/41) (9). Other studies also identified linear IgM deposits in various bullous and non-bullous skin disorders (9). Although the true pathogenic role of the IgM antibodies is unknown, the fact that IgM antibodies were present at both the initial and recurrence stages, while IgG anti-type VII collagen antibodies were not detected at the recurrence stage might suggest the pathogenic relevance of the IgM antibodies.

Previous studies reported that various autoimmune skin diseases, including AIBDs, developed skin lesions about 1 week after COVID-19 vaccination with some variation (10, 11). This case showed the recurrence only 2 days after vaccination, and therefore it may be difficult to conclude that the recurrence of the skin lesions is attributed to vaccination. Indeed, 2 days are too short for pathogenic plasma cells to produce autoantibodies.

However, there may be different mechanisms for the quick recurrence of the skin lesions. For example, the autoantibodies were persisted in the serum in this patient, and some triggers caused by vaccination might reactivate the pathogenic activity of the autoantibodies. One possibility might be similar to the mechanism in vancomycin-induced linear IgA disease, in which recurrence of the skin lesions occurred after few days of vancomycin intake, through the activation of preexisting IgA anti-collagen VII antibodies by binding of vancomycin to IgA antibodies (12).

In addition, a previous report described that the relapses of some skin diseases were seen very early (even on day 2) after COVID-19 vaccination. Therefore, we considered that this case might recur the previous autoimmune skin disease after vaccination (13).

Characteristic clinical features in our patient were prominent vesicular erythematous skin lesions on the face and severe oral mucosal lesions. Skin lesions on the face are rarely seen in both common BP and EBA (2, 3, 14–17). In addition, oral mucosal lesions are frequently seen in EBA, but are uncommon in BP (16, 17). Therefore, the prominent facial skin lesions and severe oral mucosal lesions in our patient might be cause by COVID-19 vaccination or IgM anti-BMZ autoantibodies.

EBA is a heterogeneous disease (18). In our case, the lesions are present on the mucosa and the face which is not common in normal BP. The cutaneous manifestations in EBA can be classified into two major clinical subtypes: non-inflammantory (classical or mechanobullous) and inflammatory EBA, which is characterized by cutaneous inflammation, resembling BP, linear IgA disease, MMP, or Brunsting-Perry pemphigoid (15).

The facial involvement is not uncommon, especially in the Brunsting-Perry pemphigoid-like variant of EBA, which is confined to the head and neck, was originally described in 1957 in seven patients with localized cicatricial pemphigoid (19). Similar findings have also been reported in patients with BP and EBA (20, 21). All these disorders have a common subepidermal clefting or blistering and linear IgG/complement deposition by DIF with autoantibodies against variable BMZ components such as BP180, BP230, laminin-332, and collagen VII (22).

The cutaneous manifestations of an individual EBA patients may change during the course of disease, or the same patients may present with two different forms simultaneously (14). Many EBA patients have not to lead to complete remission by the treatment (23). If disease activity cannot be controlled. Oral lesions are most commonly observed in patients with non-inflammatory EBA and those with MMP-like or LAD-like inflammatory EBA. Hence, we diagnosed her as the recurrence of non-inflammatory EBA.

In viral infections, IgM develops early stage. IgM levels increased during the first week after SARS-CoV-2 infection, peaked 2 weeks and then reduced to near-background levels in most patients (24). IgG was detectable after 1 week and was maintained at a high level for a long period (24).

In conclusion, the present case is the first case of possible EBA with IgG and IgM anti-BMZ antibodies, in which the mucocutaneous lesions were recurred 2 days after COVID-19 mRNA vaccination. Clinicians should be aware that BP-like AIBDs, including EBA and IgM pemphigoid, might be developed after COVID-19 mRNA vaccination. However, because corticosteroid treatment might have a beneficial effect on COVID-19, abrupt termination or quick dose reduction of systemic corticosteroids should be avoided in severe AIBDs (25).

## Data availability statement

The original contributions presented in the study are included in the article/supplementary material, further inquiries can be directed to the corresponding author.

## Ethics statement

The Committee of Medical Ethics of Hirosaki University Graduate School of Medicine approved the research study. The patients/participants provided their written informed consent to participate in this study. Written informed consent was obtained from the individual(s) for the publication of any potentially identifiable images or data included in this article. Written informed consent was obtained from the patient for the publication of this case report.

## Author contributions

TH and SM contributed to conception and organized the data. SY and CS contributed to collect samples. NI and HK performed the statistical analysis. SM wrote the first draft of the manuscript. TH, YM, and EA editing the manuscript. DS decided to submit report for publication. All authors contributed to manuscript revision, read, and approved the submitted version.
